# Neuroprotective Effects of the Nutraceutical Dehydrozingerone and Its C_2_-Symmetric Dimer in a Drosophila Model of Parkinson’s Disease

**DOI:** 10.3390/biom14030273

**Published:** 2024-02-24

**Authors:** Maria Dolores Setzu, Ignazia Mocci, Davide Fabbri, Paola Carta, Patrizia Muroni, Andrea Diana, Maria Antonietta Dettori, Maria Antonietta Casu

**Affiliations:** 1 Department of Biomedical Sciences, University of Cagliari, Monserrato, 09042 Cagliari, Italy; mdsetzu@unica.it (M.D.S.); pmuroni@unica.it (P.M.); diana@unica.it (A.D.); 2 Unit of Cagliari, CNR-Institute of Translational Pharmacology, Pula, 09050 Cagliari, Italy; ignazia.mocci@ift.cnr.it; 3 Unit of Sassari, CNR-Institute of Biomolecular Chemistry, 07100 Sassari, Italy; davidegaetano.fabbri@cnr.it (D.F.); paola.carta@cnr.it (P.C.)

**Keywords:** Parkinson’s disease, nutraceutical compounds, dehydrozingerone, hydroxylated biphenyls, natural compounds, *Drosophila melanogaster*, LRRK2, neuroprotection

## Abstract

Parkinson’s disease (PD) is a neurodegenerative disorder characterized by the loss of dopaminergic neurons responsible for unintended or uncontrollable movements. Mutations in the leucine-rich repeat kinase 2 locus contribute to genetic forms of PD. The fruit fly *Drosophila melanogaster* carrying this mutation (LRRK2-Dm) is an in vivo model of PD that develops motor impairment and stands for an eligible non-mammalian paradigm to test novel therapeutic approaches. Dehydrozingerone (DHZ) is a natural phenolic compound isolated from ginger and presents anti-inflammatory, antioxidant and neuroprotective properties, making it a potential therapeutic target for PD. We administered DHZ and its C_2_-symmetric dimer (DHZ-DIM) at 0.5 and 1 mM for 14 and 21 days in the LRRK2-Dm, with the aim of assessing changes in rescuing motor behavior, brain dopaminergic neurons, mitochondria and synapses (T-bars). The shorter treatment with both molecules revealed efficacy at the higher dose, improving climbing behavior with a prevention of dopaminergic neuronal demise. After 21 days, a recovery of the motor disability, dopaminergic neuron loss, mitochondrial damage and T-bars failure was observed with the DHZ-DIM. Our data indicate that the DHZ-DIM exerts a more potent neuroprotective effect with respect to the monomer in LRRK2-Dm, prompting further investigation of these compounds in rodent models of PD.

## 1. Introduction

Parkinson’s disease (PD) is an age-related neurodegenerative disorder with typical manifestation of motor symptoms that includes bradykinesia, rigidity, postural instability, and tremor associated with several non-motor symptoms, namely, cognitive impairment, depression, sleep disturbance and olfactory deficits [1,2,3,4,5]. A pathological hallmark of the disease is the loss of dopaminergic neurons in the substantia nigra, though the processes underlying dopaminergic cell death remain unclear, as well as the exact etiology. However, some biochemical mechanisms are recognized as contributors to the neuropathology: first of all, an abnormal and toxic intracellular accumulation and aggregation of misfolded proteins, such as α-synuclein and parkin, which converge into the core of Lewy bodies. Moreover, neuroinflammation characterized by reactive microgliosis, oxidative stress caused by overproduction of reactive oxygen species, in combination with reactive metabolites of dopamine, and mitochondrial dysfunction are significantly present [6,7,8].

With regard to available therapies for PD, an effective preventive neuroprotective or disease-modifying cure is currently lacking, highlighting the urgent need for novel drugs or alternative strategies at least to halt the progression of the disease. In recent decades, many studies have adopted a more holistic approach based on metabolic amelioration achieved by specific dietetic programs, such as possible preventive therapy for neurodegenerative diseases. As a matter of fact, many nutraceuticals and food-derived bioactive compounds, by virtue of their intrinsic properties, could have a dramatic role in reducing the risk factors for the arising of chronic neurodegenerative diseases. Focused emphasis has been given to the use of polyphenols, present in most vegetables, e.g., flavonoids, phenolic acids or curcuminoids, as therapeutic natural compounds against inflammation, neurodegeneration, and oxidative stress [9,10,11].

**Figure 1:**
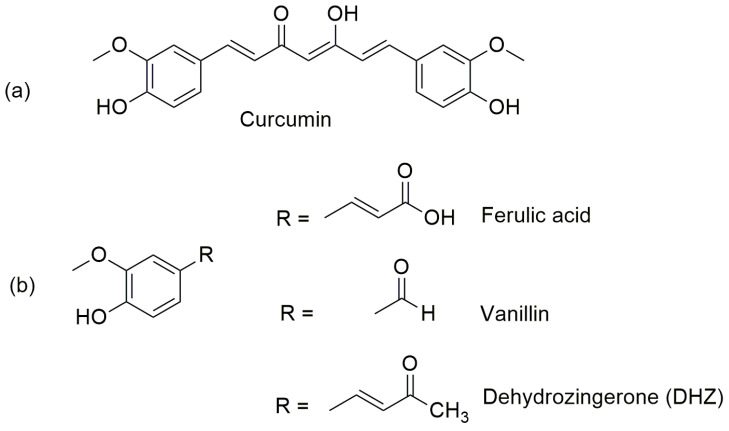
Chemical structures of (**a**) curcumin; (**b**) ferulic acid, vanillin and dehydrozingerone (DHZ).

Curcumin (Figure 1a), also known as diferuloylmethane, is the active component of the *Curcuma longa* (*Zingiberaceae* family) rhizome. This rhizome meets large appreciation as a spice in Indian curries and has garnered specific attention for its wide range of pharmacological activities [12,13,14]. Many studies in vitro and in vivo showed that curcumin possesses neuroprotection properties [15,16]. Moreover, it has been reported that curcumin is able to suppress PD-like phenotypes in flies [17,18,19].

Unfortunately, the pharmacological potential of curcumin is widely restricted because of its poor bioavailability, due to its chemical instability and rapid metabolic degradation into ferulic acid, vanillin and dehydrozingerone (DHZ) at physiological pH (Figure 1b) [17]. Therefore, it makes sense to explore the development of curcumin derivatives with enhanced bioavailability with consequent translational benefits to hinder PD by identification and production of more potent compounds in the context of phytotherapeutic options.

As mentioned above, DHZ, a structural half analogue of curcumin (CUR), is a natural phenolic compound extracted from ginger (*Zingiber officinale*) rhizome that exhibits enhanced solubility and stability in water compared to CUR. This property gives DHZ a tremendous advantage for biomedical applications where water solubility and stability are crucial factors.

It has been demonstrated that DHZ exhibits many biological activities and pharmacological properties, including anti-inflammatory, antioxidant, anti-obesity, anti-cancer, tyrosinase-inhibitory, neuroprotective, antidepressant and anti-fungal effects [18,19,20,21,22,23,24,25,26,27,28].

Often, hydroxylated biphenyls, have higher biological activities with respect to the corresponding monomer. Hydroxylated biphenyls are widely distributed in the plant kingdom and play an important role in biosystems due to their unique pharmacophore structure that is made up of two aromatic rings bridged by a single C–C bond. The high level of specificity of the hydroxylated biphenyls scaffolds with the catalytic domain of different proteins suggests that this moiety could be an effective scaffold for the discovery and design of new protein targets [29,30,31]. Consequently, the hydroxylated biphenyl framework offers an ideal molecular structure for structural modifications in the development of potential drug candidates [32].

In prior studies, we established that both DHZ and its symmetric dimer (DHZ-DIM) exhibit protective effects against lipid autoxidation [33], which is an important factor in the development of neurodegenerative disorders such as Alzheimer’s and Parkinson’s disease. Furthermore, DHZ-DIM exerted a potent anti-inflammatory, antioxidant, and antithrombotic activity on endothelial cells in combination with antiaggregating and cytoprotective properties, as demonstrated by its ability to partially inhibit the aggregation process of alpha-synuclein [34,35]. For these reasons, DHZ and DHZ-DIM could represent interesting candidates to reverse the symptoms of PD.

Although most PD cases seem sporadic, genetic factors may play a role in favoring the disease, and mutations in several specific genes have been related to familiar forms of PD. Among them, mutations in the leucine-rich repeat kinase 2 (LRRK2) gene have been correlated with late-onset autosomal dominant PD. Pathological mutations of LRRK2 have been found not only in 13% of familial forms but also in approximately 1–2% of idiopathic PD cases [36,37]. Furthermore, familial forms related to mutations in the LRRK2 gene show clinical symptoms indistinguishable from idiopathic forms of PD [38].

LRRK2 is a protein widely expressed within different brain areas, such as the cortex, striatum, hippocampus, cerebellum, and substantia nigra [39,40]. It is involved in GTPase and kinase activities associated with signal transduction cascades for synaptic vesicle trafficking, mitochondrial metabolism, and autophagy [41,42]. LRRK2 holds a double enzymatic core in the N-terminal and the C-terminal WD40 domain [43]. In particular, the coding variants G2019S, diffuse in Caucasian individuals [44], and G2385R, present in Asian population [45], in the WD40 domain, resulted in about 50% loss of kinase activity leading to a partial loss of function of LRRK2 [46]. These missense substitutions double the risk of developing PD [47,48,49].

Regarding the PD genetic approach, *Drosophila melanogaster* (Dm), commonly known as the fruit fly, is a powerful translational animal model for studying neurodegenerative diseases, as it carries nearly 75% homology with human disease genes [50]. Moreover, Dm has several advantages compared to mammalian models, from expandable population, quick life cycles, and easy genetic manipulation to low maintenance costs and less rigid ethical concerns. Notably, most of the genes implicated in familial forms of PD have an evolutionary counterpart in Dm [51,52]. Therefore, Dm carrying the LRRK2 loss-of-function mutation in the WD40 domain [53] develops the essential traits of the pathology, such as motor impairment, dopaminergic neuronal cell loss and mitochondrial abnormalities [54,55,56], providing precious information regarding PD pathophysiology mechanisms and a bona fide tool to firstly test novel therapeutic approaches to the disease.

Thus, the aim of this paper was to investigate the neuroprotective potential of DHZ and DHZ-DIM, prepared following new sustainable procedures, carrying out the reactions under microwave irradiation. These two compounds were tested on the Drosophila PD strain LRRK2 loss of function (LRRK) compared to wild type (w1118) in terms of physiological and brain morphological parameters that are severely compromised in mutant flies, such as longevity, motor activity, integrity of brain dopaminergic neurons, brain mitochondria and synapse abnormalities. After treatment with both molecules at doses of 0.5 and 1 mM and vehicle for 14 and 21 days after eclosion, there was a significant amelioration of motor performance, and prevention of dopaminergic neuron loss, mitochondrial damage, and synapse (T-bar) failure in LRRK mutants. Specifically, our data indicate that the DHZ-DIM exerts a more potent neuroprotective effect with respect to the monomer in this fruit fly PD model suggesting that these curcumin-related compounds could be promising medicaments for novel therapeutic scenarios toward LRRK2-linked PD.

## 2. Materials and Methods

### 2.1. General

Reagents were obtained from Sigma Aldrich (Munich, Germany) and were used without further purification. Microwave reactions were carried out on the MW instrument CEM-Discover SP MW (Matthews, NC, USA). 1H-NMR and 13C-NMR spectra were recorded in CDCl3 at 600 and 150 MHz, respectively, with a 600 MHz NMR spectrometer Bruker Avance III HD (Palo Alto, CA, USA). Chemical shifts are given in ppm (δ); multiplicities are indicated by s (singlet), d (doublet), or dd (doublet of doublets). Elemental analysis was performed using an elemental analyzer model 240 C Perkin Elmer (Waltham, MA, USA). Flash chromatography was carried out with silica gel 60 (230–400 mesh) VWR (Radnor, AF, USA) eluting with the appropriate solution in the stated *v:v* proportions. Reaction was monitored by analytical thin-layer chromatography (TLC) with 0.25 mm-thick silica gel plates 60 F 254 Sigma Aldrich (Munich, Germany). Melting point was determined on a 530 apparatus Büchi (Flawil, Switzerland) and is uncorrected. The purity of new compounds was judged to be >98% by 1H-NMR spectral determination.

### 2.2. Fly Stocks

Adult wild-type (WT; w1118) and LRRKWD40 (LRRK)-mutant (LRRKex1, #34750, from Bloomington Stock Center) Dm males were used. After emergence from pupae, WT or LRRK-mutant males were separated from females. WT and mutant flies were reared on a standard cornmeal–yeast–agar medium in controlled environmental conditions (24–25 °C; 60% relative humidity; light/dark = 12/12 h). For the treatment, groups of mutant and WT flies were reared on a standard medium supplemented with two concentrations of DHZ and DHZ-DIM (0.5 and 1 mM) for 14 and 21 days. The confirmation of DHZ and its dimer ingestion was assessed by the visualization of red (DHZ) and blue (DHZ-DIM) abdomen due to color-marked enriched medium (Figure S1).

### 2.3. Drugs

DHZ and DHZ-DIM were dissolved in DMSO (final concentration 0.5%) and added to the diet at the concentrations reported.

### 2.4. Climbing Assay

The climbing assay (negative geotaxis assay) was used to assess locomotor ability [57] in WT and LRRK mutants treated for 14 or 21 days with DHZ, DHZ-DIM or vehicle. Cohorts of at least 30 flies from each group, in three independent experiments, were subjected to the assay. Flies were placed individually in a vertically positioned plastic tube (length 10 cm; diameter 1.5 cm) and tapped to the bottom. Climbing time (s) was recorded upon crossing a line drawn at 6 cm from the bottom. The number of flies that could climb unto or above this line within 10 s was recorded and expressed as a percentage of the total flies tested. Data are expressed as the average ± standard error of the mean (SEM) from three experiment replications.

### 2.5. Survival Curves

In accordance with previous reports [58], WT and LRRK male flies were separated from females after emergence from pupae under CO_2_ anesthesia. Cohorts of 60 flies from each group were collected in groups of 10–15 in vials containing a standard diet with drugs at 1 mM or VEH, monitored daily at 25 °C and changed with frequency throughout adult life. Data were collected from eclosion to death. For mortality analysis, Kaplan–Meier survival curves and statistical comparisons using the log-rank (Mantel–Cox) test and Gehan–Breslow–Wilcoxon test were utilized.

### 2.6. Immunohistochemistry

Six to ten flies from each experimental group were selected to perform free-floating fluorescent immunostaining for tyrosine hydroxylase (TH). Animals were anesthetized on ice, and brains were rapidly dissected and fixed in 4% paraformaldehyde in phosphate-buffered saline (PBS) for 2 h. Brains were then incubated with the TH rabbit primary antibody (1:100; AB 152 Millipore Corporation, Billerica, MA, USA) and 10% normal donkey serum in PBS + 0.3% Tween 20 (PBST), at 4 °C for 72 h. After rinsing, the brains were incubated with a donkey anti-rabbit Alexa Fluor 594 secondary antibody (1: 200 Jackson ImmunoResearch Europe, Newmarket, UK) and 10% normal donkey serum in PBST at 4 °C for another 72 h. Subsequently, the brains were mounted on glass slides, coverslipped with Vectashield and analyzed under a fluorescence-spinning disk confocal microscope (Crisel Instruments Rome, Italy). The brains were scanned through Z-stacks (63X objective, stack thickness 0.5 µm), and the number of TH-positive neurons of different clusters (PPL1, PPL2, PPM1/2, PPM3) in both hemispheres was counted via ImageJ software Fiji 1.46r (National Institutes of Health, Bethesda, MD, USA).

### 2.7. Electron Microscopy Analysis

The electron microscopy studies were performed in strict accordance with the general methodological procedures indicated by Casu et al. (2020) [56].

Briefly, flies from each experimental group (n = 5), were anesthetized on ice, and brains were rapidly dissected and fixed in a mixture of 1% paraformaldehyde and 1.25% glutaraldehyde in 0.15 M cacodylate buffer for 2 h. Brains were then post-fixed with 1% osmium tetroxide for 1 h and stained overnight with 0.5% uranyl acetate at 4 °C. After dehydration in a graded acetone series, brains were embedded in EPON resin. To identify the protocerebrum, where the dopaminergic posterior clusters reside, 1 µm semi-thin coronal sections of the whole brain were stained with toluidine blue. Ultrathin sections (90 nm) cut with a Reichert Supernova ultramicrotome were counterstained with uranyl acetate and lead citrate and observed under a JEOL JEM 1400 Plus electron microscope equipped with a CCD camera at an acceleration voltage of 80 kV.

For morphometric analysis, the mitochondria (total number), the percentage of mitochondria with swollen cristae (percentage of mitochondria displaying swollen cristae versus total mitochondria with discernible cristae) and the T-bar density were analyzed in the unitary area (25 µm^2^) in the protocerebrum. Thirty to forty unitary fields were evaluated for each brain. In total, about 17,500 mitochondria and 4000 T-bars were randomly sampled on 792 non-overlapping micrographs at a magnification of 8000×. Swollen cristae were considered when the distance between two contiguous membranes of one crista doubled the average crista size. T-bars were unambiguously identified at presynaptic active zones by the presence of T-shaped electron-dense projections.

### 2.8. Statistics

Data are presented as means ± SEM. Two group comparisons have been analyzed by factorial two-way ANOVA with the strain and treatment as between-group factors. Before performing the analyses, datasets were checked for normal distribution using the Shapiro–Wilk test and for homogeneity of variances between the experimental groups with Bartlett’s test. When the normal distribution of data and homogeneous variances across the experimental groups were obtained in all datasets, the factorial ANOVA was applied.

In all the other cases, when the transformation data did not reveal homogeneity of variances, non-parametric analysis by the Kruskal–Wallis comparisons test was performed. When parametric two-way ANOVA revealed statistically significant interactions, sources of significance were ascertained by pairwise post hoc analyses using the HSD Tukey’s test. For mortality analysis, Kaplan–Meier survival and statistical comparisons with Gehan–Breslow–Wilcoxon tests were used. Statistical analyses were all carried out with PRISM, GraphPad 8.0.1 Software (2018), with the significance level set at *p* < 0.05.

## 3. Results

### 3.1. Chemistry

**Scheme 1:**
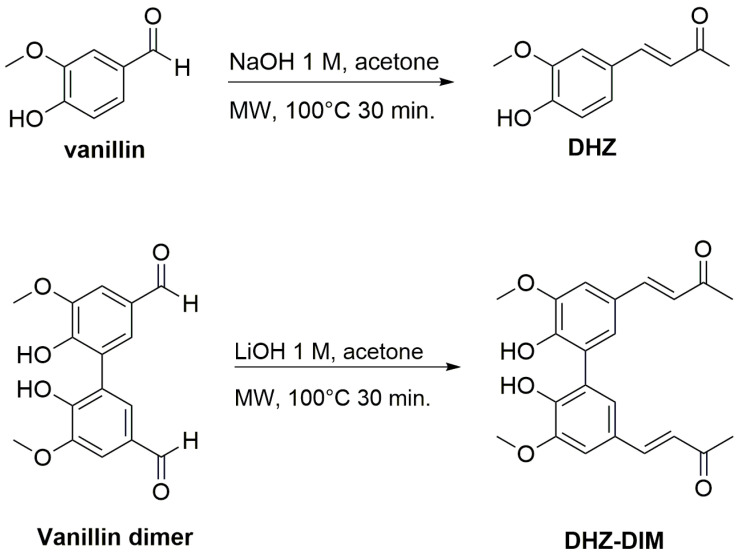
Synthesis of dehydrozingerone (DHZ) and its dimer (DHZ-DIM).

DHZ and DHZ-DIM were synthesized with comparable yields and purity, employing a method previously outlined by our group [34]. Notably, we followed more sustainable procedures, utilizing microwave irradiation to significantly reduce the reaction time from 12 h to just 30 min. DHZ was prepared by Claisen–Schmidt condensation reaction of vanillin and acetone in the presence of 1 N NaOH as base (90% yield). DHZ-DIM was prepared under the same conditions, starting from vanillin dimer [35] and acetone in the presence of 1 N LiOH as base (83% yield) (Scheme 1).

#### 3.1.1. Synthetic Procedures

##### [(E)-4-(4-Hydroxy-3-methoxyphenyl) but-3-en-2-one] (DHZ)

An aqueous solution of NaOH 1 N (5.2 mL, 5.2 mmol) was added to a vanillin solution (0.2 g, 1.3 mmol) in acetone (7 mL). The reaction mixture was stirred under MW irradiation at 100 °C for 30 min. The solvent was then roto-evaporated and 10% HCl was cautiously added. The obtained heterogeneous solution was extracted with ether, and the organic phase dried over Na_2_SO_4_ and evaporated to afford a brown solid compound. The crude material was finally purified by flash chromatography using CH_2_Cl_2_ as eluent to give DHZ as yellow solid (0.23 g, 90%): mp 126–127 °C; 1H NMR δ 2.39 (s, 3H), 3.85 (s, 3H), 5.99 (bs, 1H), 6.52 (d, J = 16.0 Hz, 1H), 6.90 (d, J = 8.0 Hz, Ar, 1H), 7.00 (d, J = 1.6 Hz, Ar, 1H), 7.06 (dd, J = 1.6, 8.0 Hz, Ar, 1H), 7.40 (d, J = 16.0 Hz, 1H); 13C NMR δ 27.29, 56.93, 109.30, 114.80, 123.94, 124.99, 126.95, 143.76, 146.84, 148.26, 198.46; Anal. Calcd for C11H12O3: C, 68.74; H, 6.29; Found: C, 68.93; H, 6.41.

##### [(3E,3′E)-4,4′-(6,6′-Dihydroxy-5,5′-dimethoxy-[1,1′-biphenyl]-3,3′-diyl) bis(but-3-en-2-one)] (DHZ-DIM)

An aqueous solution of LiOH 1 N (4.0 mL, 4.0 mmol) was added to vanillin dimer (0.2 g, 0.66 mmol) in acetone (5 mL). The reaction mixture was stirred under MW irradiation at 100 °C for 30 min. The solvent was then roto-evaporated and 10% HCl was cautiously added. The obtained precipitate was filtered, washed with water and dried to afford a DHZ-DIM as yellow solid compound (0.18 g, 83%): mp 242–243 °C; 1H NMR δ 2.35 (s, 6H), 3.99 (s, 6H), 5.28 (bs, 2H), 6.61 (d, J = 16.0 Hz, 2H), 7.1 (d, J = 2.0 Hz, Ar, 2H), 7.15 (d, J = 2.0 Hz, Ar, 2H), 7.47 (d, J = 16.0 Hz, 2H); 13C NMR δ 27.35, 56.20, 108.67, 123.59, 125.28, 125.44, 126.60, 143.52, 145.45, 147.36, 198.28; Anal. Calcd for C_22_H_22_O_6_: C, 69.10; H, 5.80; Found: C, 69.47; H, 5.70.

### 3.2. DHZ and DHZ-DIM Prevent Motor Impairment in LRRK: Climbing Test

**Figure 2:**
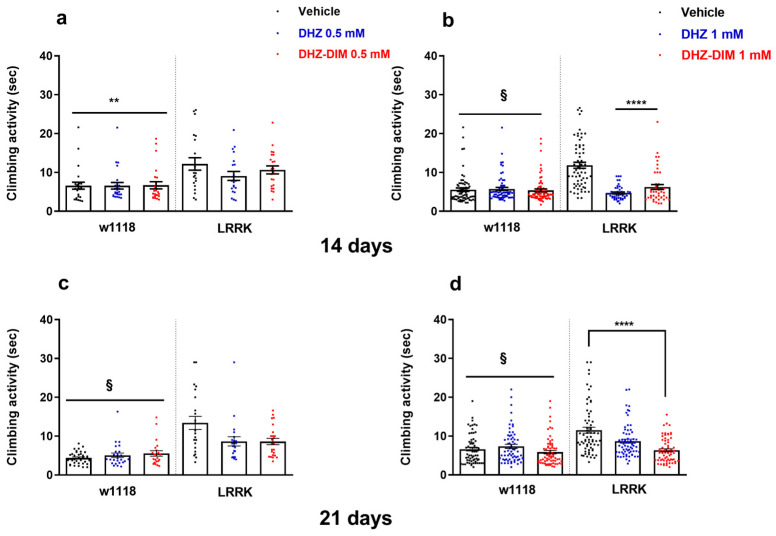
Effect of DHZ and DHZ-DIM at 0.5 mM and 1 mM on the climbing behavior parameters in both LRRK and w1118 WT flies and their respective control groups at 14 (**a**,**b**) and 21 days (**c**,**d**) of treatment. ^§^ and **** *p* < 0.0001 vs. LRRK vehicle; ** *p* < 0.01 vs. LRRK vehicle.

Groups of LRRK and WT flies received both DHZ or DHZ-DIM at the two doses of 0.5 and 1 mM in their diet for 14 and 21 days to evaluate the effect of both molecules on locomotor ability. The climbing activity and the percentage of flies reaching the target within 10 s were measured. Remarkably, after 14 days of both molecules’ treatment, a significant improvement in the climbing behavior of LRRK flies was observed only with the highest dose of 1 mM if compared to the LRRK vehicle (*p* < 0.0001 Figure 2a,b). On the other hand, at the same dose (1 mM), only DHZ-DIM was able to induce a recovery of the motor disability after 21 days of treatment (*p* < 0.0001). The administration of the same drugs at both concentrations did not significantly affect in the w1118 flies (Figure 2a–d).

**Figure 3:**
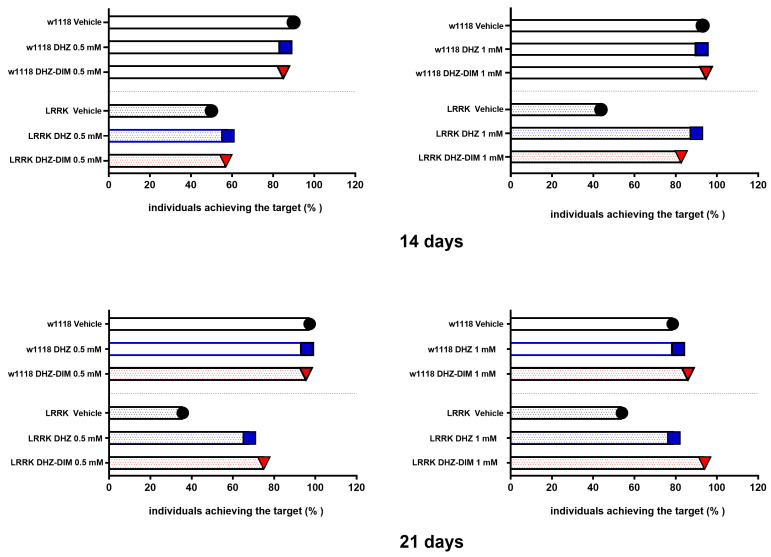
Percentage of w1118 and LRRK individuals reaching the target within 10 s.

In the search for an additional parameter of motor ability, we counted the percentage of WT and LRRK flies completing the climbing test within 10 s (Figure 3). The resulting relative percentages confirmed the improvement in mutants treated for 14–21 days, regardless of DHZ and DHZ-DIM at the concentrations of 0.5 and 1 mM. In addition, the DHZ-DIM was more effective at the higher 1 mM dose on both treatment timelines.

### 3.3. DHZ and DHZ-DIM Extended Longevity

**Figure 4:**
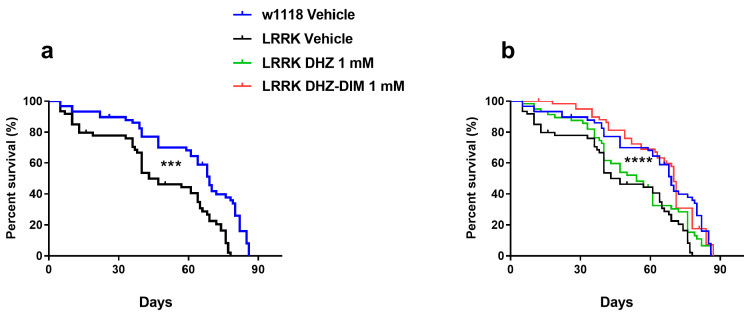
Life span. (**a**): reduced life span in LRRK compared to w1118 flies, both treated with vehicle; (**b**): survival rate observed in DHZ- and DHZ-DIM-treated LRRK flies at 1 mM compared to WT vehicle. Cumulative survival curve data are expressed as means ± SEM. *** *p* < 0.001 indicates significant difference between LRRK vehicle and WT vehicle. **** *p* < 0.0001 LRRK vehicle vs. LRRK treated with DHZ-DIM (Gehan–Breslow–Wilcoxon test).

Assessment of fly life span using survival curves allowed monitoring of the effect of drugs on Dm survival throughout adulthood. LRRK displayed a shorter life span compared to WT w1118 (*p* < 0.0001, Figure 4a) flies, since mutant flies encountered a dramatic decay in their survival rate (about 50%) at 35–40 days after enclosure (with median survival at 47 days). In an attempt to prolong the LRRK life span, aimed at verifying to what extent the studied molecules were ad hoc effective, mutant flies were supplied with DHZ and DHZ-DIM at the higher concentration of 1 mM and compared with vehicle-treated flies. Both compounds significatively extended longevity of LRRK (*p* < 0.0001, Figure 4b) flies, although flies treated with DHZ-DIM showed a lower decay in the survival rate with the median survival about 70 days with respect to DHZ (around 54 days). Moreover, a small group of LRRK flies (about 20%) treated with the dimer achieved a survival rate at 80 days like w1118 flies.

### 3.4. DHZ and DHZ-DIM Prevent the Loss of Dopaminergic Neurons

**Figure 5:**
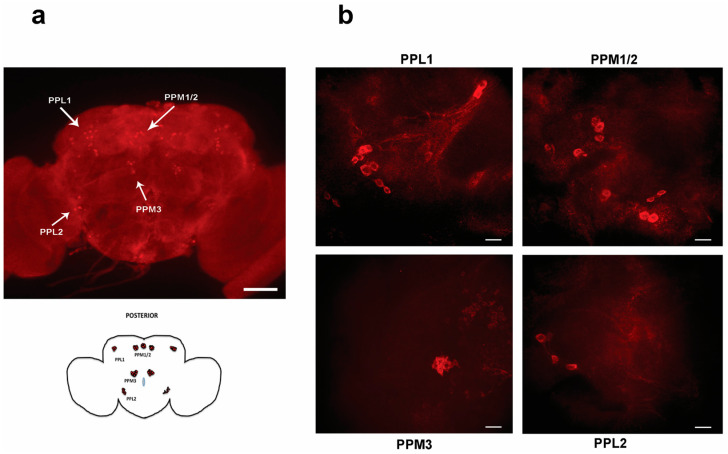
Representative image stacks showing the entire brain ((**a**): 10× scale bar: 100 µm) and the dopaminergic brain neurons in all posterior clusters ((**b**): 63×, scale bar: 10 µm).

After the climbing behavioral test, flies were euthanized to evaluate the effect of DHZ and DHZ-DIM treatment on TH-positive neurons in the brain posterior dopaminergic clusters (PPL1, PPL2, PPM1/2 and PPM3 (Figure 5)). Since both compounds displayed a maximal behavioral effect at 1 mM concentration, only brains from flies exposed for 14 and 21 days to the above concentration of DHZ and DHZ-DIM were processed for TH immunohistochemistry.

**Figure 6:**
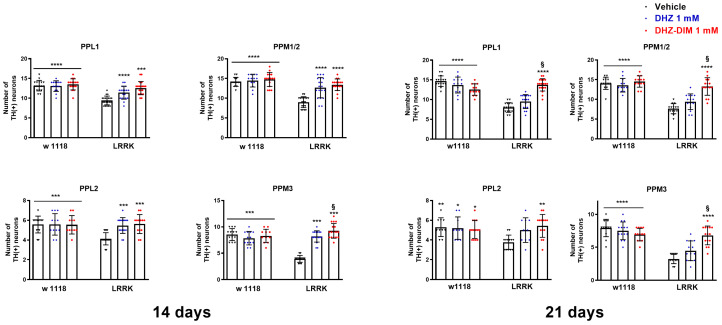
DHZ and DHZ-DIM prevent the loss of dopaminergic neurons. Both compounds at 1 mM significantly prevented the loss of dopaminergic neurons in all posterior clusters at 14 days of treatment, whereas at 21 days, a significant prevention of dopaminergic neuron loss was observed only with the DHZ-DIM treatment. * *p* < 0.05, ** *p* < 0.01, *** *p* < 0.001 and **** *p* < 0.0001 indicate significant differences between LRRK2 vehicle and LRRK2 treated with DHZ, DHZ-DIM or vehicle and treated WT groups. ^§^ *p* < 0.0001 LRRK treated with DHZ-DIM vs. LRRK treated with DHZ.

The quantitative analysis of TH immunofluorescent neurons showed that both compounds at 1 mM significantly prevented the loss of dopaminergic neurons in all four posterior clusters after 14 days of treatment (*p* < 0.0001). However, at 21 days, a significant prevention of dopaminergic neuronal demise was surprisingly detected only with the DHZ-DIM treatment (*p* < 0.0001) (Figure 6).

### 3.5. DHZ-DIM Is More Effective Than Monomer in Preventing the Mitochondrial Damage and the Loss of T-Bars in LRRK2 Drosophila

**Figure 7:**
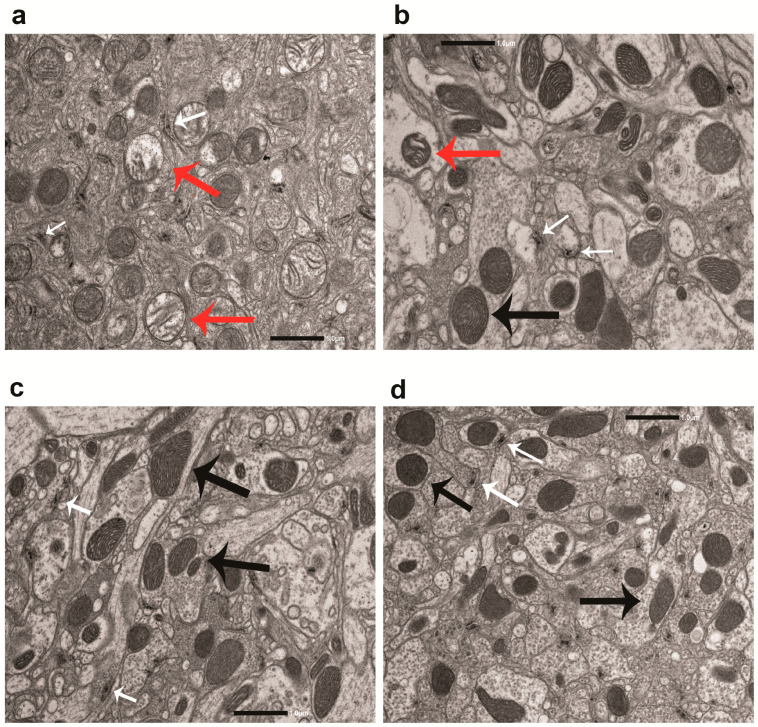
Representative images of mitochondria (8000X) acquired from the protocerebrum of LRRK and w1118 flies treated with vehicle (**a**–**d**), LRRK treated with DHZ (**b**) and DHZ-DIM (**c**) for 21 days. Red arrows: mitochondria with swollen cristae; black arrows: mitochondria with normal cristae; white arrows: T-bars.

Transmission electron microscopy (TEM) analysis was conducted on LRRK and WT brain flies treated with vehicle, DHZ or DHZ-DIM at the dose of 1 mM for 21 days, since immunohistochemical results showed that both compounds elicited the maximal effects at 1 mM concentration, with particular reference to the latest time point treatment. As previously described, ultrastructural brain morphology showed axons and terminal boutons, mitochondria and T-bar presynaptic densities (Figure 7a–d). In particular, in vehicle-treated LRRK brain flies, several mitochondria displaying swollen cristae were present (Figure 7a).

**Figure 8:**
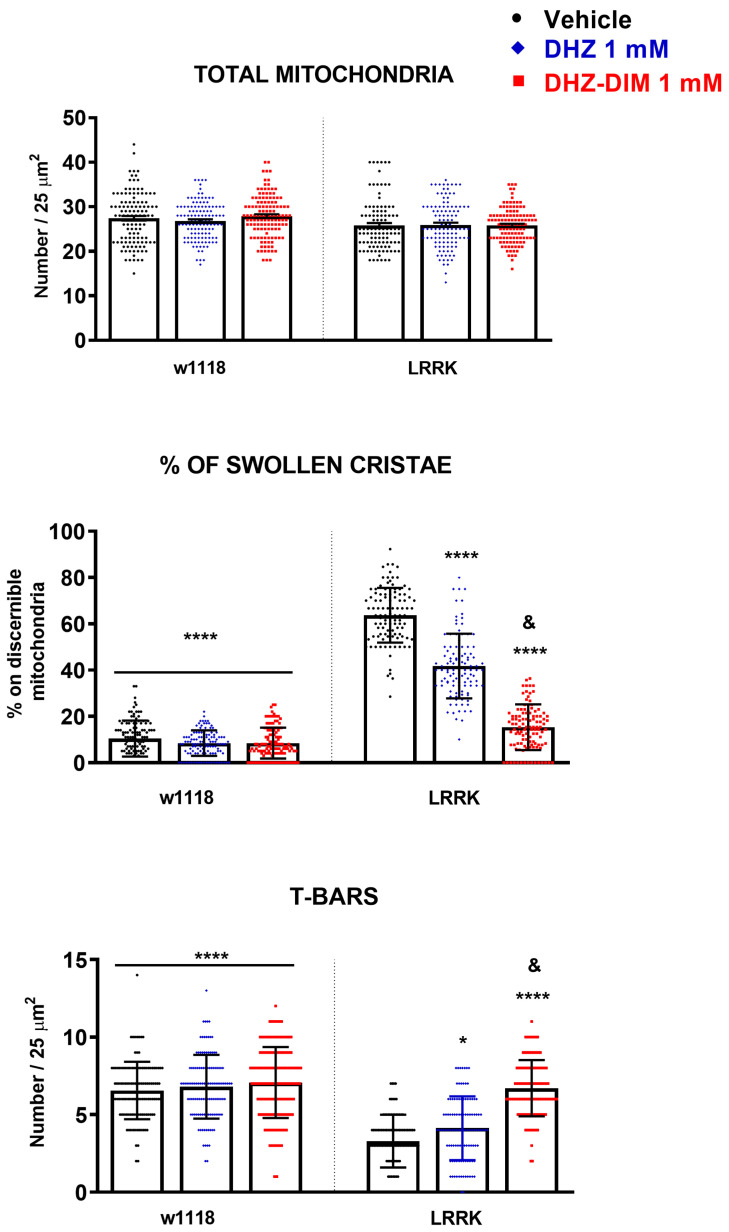
DHZ and DHZ-DIM effect on the number of total mitochondria, percentage of cristae and T-bars in the protocerebrum of LRRK after 21 days of treatment. * *p* < 0.05 and **** *p* < 0.0001 indicate significant differences between LRRK vehicle and LRRK treated with DHZ, DHZ-DIM or vehicle and treated WT groups. ^&^ *p* < 0.0001 LRRK treated with DHZ-DIM vs. LRRK treated with DHZ.

The morphometric analysis did not reveal any difference in the number of total mitochondria between control and LRRK treated with DHZ or DHZ-DIM (Figure 8). The administration of both substances clearly decreased the occurrence of mitochondria with swollen cristae compared with vehicle administration, (*p* < 0.0001) and more relevantly, in accordance with other morphological and functional parameters, the treatment with DHZ-DIM was more worthwhile than DHZ in avoiding the formation of swollen mitochondria (Figure 8). Similarly, using both compounds, a significant increase in the number of T-bar in the presynaptic bouton active zones compared to vehicle treatment was observed (*p* < 0.01; Figure 8), but the contribution of the dimer was definitely more impactful. By contrast, the exposure of WT flies to both monomer and dimer changed neither mitochondria number, morphology, nor the number of T-bars.

## 4. Discussion

In the present study, we investigated the neuroprotective activity of the two curcumin derivatives DHZ and its C_2_ dimer in a transgenic Drosophila model of PD. We improved the process sustainability of DHZ and DHZ-DIM synthesis by carrying out the reactions under microwave (MW) irradiation. As matter of fact, MW technology presents numerous advantages in various chemical synthesis processes. This efficiency is attributed to the ability of MW in heating the reaction mixture both rapidly and uniformly, promoting faster and more complete chemical transformations compared to conventional methods. Moreover, this technology minimizes the risk of hazardous reactions or the release of volatile substances, contributing to a safer working environment. The utilization of MW aligns with the broader goals of sustainability, promoting energy efficiency and reducing overall environmental impact. To our knowledge, this is the first study evaluating DHZ and DHZ-DIM as neuroprotective agents using an in vivo model of PD. Our data demonstrate that exposure to both compounds prevented motor deficits and protected against the progressive loss of dopaminergic neurons. The neuroprotective efficacy of the DHZ and DHZ-DIM could be related to their antioxidant action, since in previous studies, we found that both DHZ and its symmetric dimer (DHZ-DIM) exhibit protective effects against lipid autoxidation when used in combination with conventional antioxidants [33]. The antioxidant activity of DHZ-DIM is also associated with antiaggregating and cytoprotective properties, ascertained by its ability to partially inhibit the aggregation process of α-synuclein [35].

The methodological approach to the morphological study has taken into account that in the Drosophila brain, DA neurons are organized in distinct bilateral symmetric clusters with projections onto specific brain areas [59,60]. These dopaminergic neurons of posterior clusters analyzed in this study, such as PPL1, PPM1/2 and PPL2, innervate distinct regions of the mushroom bodies that are implicated with learning and memory [61]. Moreover, PPM3 neurons innervate the central complex, which is the area related to the control of motor activity.

Therefore, the neuronal rescue of the above anatomical circuits upon DHZ and DHZ-DIM directly correlates with the protective role for contrasting those brain areas dramatically affected by the progression of PD. In this context, DA neurons’ degeneration in PD is further boosted by oxidative stress mechanisms also involving DA facing rapid oxidation. The dopamine autoxidation produced dopamine quinones and free radicals. Moreover, the cyclization of dopamine quinones forms aminochrome, which generates superoxide and down-regulates antioxidative nicotinamide adenine dinucleotide phosphate (NADPH) [59,60]. The susceptibility of the brain to oxidative stress is augmented by various factors, such as high oxygen demand, higher rates of oxidative metabolism and lower levels of protective antioxidants.

This critical scenario is further worsened in the PD genetic models, where LRRK2 mutation caused increased generation of ROS and cell toxicity. A proof of concept has been offered by a recent in vitro work showing that LRRK2 knockout provides resistance to oxidative stress and apoptosis, suggesting LRRK2 as a proapoptotic kinase [62]. Moreover, previous studies demonstrated that deletion of the WD40 domain prevents autophosphorylation [63,64], and on the other hand, the G2385R polymorphism in the WD40 domain, expressed in our drosophila PD model, increases the sensitivity of cells to hydrogen peroxide, suggesting a pro-apoptotic mechanism [65].

Moreover, since recent literature data presume the association of LRRK mutation with autophagy and lysosomal dysregulations [66], this appealing and convincing hypothesis deserves future deeper investigation, especially due to the neuroprotective role of DHZ and DHZ-DIM through modulation of these biological activities.

Considering that our compounds act as antioxidants, the observed neuroprotective ability on dopaminergic neurons matched well with the prevention of motor impairment in mutant flies.

It is important to note that after 14 days of treatment, DHZ and its dimer both effectively hindered the symptoms of Parkinson’s diseases in LRRK flies, but only after a longer treatment of 21 days. The DHZ dimer is superior to the monomer in avoiding motor impairment and loss of dopaminergic neurons. These results are reasonably related to the aging progression that—by wide scientific consensus—is one of the major risk factors for developing PD. This is also suggested by the fact that there are many common features between PD and normal aging [67], including protein aggregation [47], increased oxidative stress [68], decreased mitochondrial function [69], dysfunction of the proteasome [70], and impairment of autophagy [71]. Therefore, in aged parkinsonian flies, the DHZ-DIM demonstrates greater efficacy compared to the monomer. This enhanced DHZ-DIM activity could be ascribed to the differences in the chemical structure and lipophilicity between the two molecules, and it makes sense to draw the hypothesis that the DHZ-DIM has a higher ability to cross the cell membrane and to interact with cell components more efficiently than the corresponding monomer. The superior ability of DHZ-DIM in protecting lipids from autoxidation has also been demonstrated, and additionally, its higher antioxidant properties and reactivity when compared to its corresponding monomer [33,72].

In our study, DHZ-DIM has been proven to be more decisive than monomer also in extending longevity of parkinsonian flies. This finding replicates the conclusion of several studies showing that dietary supplementation with compounds rich in polyphenols such as Avocado Persea americana, grape and grape seed extracts, gallic acid and very high doses of curcumin enhanced the life span of Drosophila models of Parkinson disease [73,74,75]. Polyphenols can delay oxidative reactions in cells by rapidly donating protons to radicals or by forming complexes with pro-oxidant metals. Furthermore, polyphenols can interact with receptors or enzymes in signal translation, promoting an antioxidant condition [10,76].

The neuroprotective effect on motor improvement and brain dopaminergic neurons of our compounds fits very well with the reduced mitochondrial damage in LRRK brain flies detected in our investigation, confirming previous findings related to the presence of damaged mitochondria in LRRK mutant flies, as indicated by multiple dilated cristae [56]. In that regard, oxidative phosphorylation within the mitochondria accounts for the majority of ATP production in neurons required for the transmission of nerve impulse. Various studies have suggested that mitochondrial dysfunction can contribute to increased levels of oxidative stress and can affect neuronal degeneration [77,78,79]. In postmortem studies, high oxidation of proteins and DNA stimulated parallel levels of lipid peroxidation such that reduction in glutathione was found in the substantia nigra in PD patients [80]. Moreover, mitochondrial complex I inhibition has been observed in PD patients, suggesting that the increased presence of ROS through complex I inhibition is one of the major contributors to DAergic neuronal cell death in PD, reinforcing the concept that this type of stress is dramatically involved in the pathology [1]. On top of such considerations, it cannot be overlooked that several genetic mutations, including LRRK2, are linked to mitochondrial dysfunction in PD pathogenesis. For example, LRRK2 interacts with the mitochondrial fusion proteins and mitochondrial outer membrane proteins [81,82]. LRRK2 mutations determined alterations in mitochondrial fusion and fission mechanisms, mitophagy and in mitochondrial DNA damage [6,83], all elements that increase ROS production, inhibition of peroxidase activity, and a consequent increment in oxidative stress [84].

The association of mitochondrial dysfunction and production of ROS represents a potential target for treating PD. Mitochondria-targeted antioxidants and flavonoids such as alpha-lipoic acid, hesperidin (flavanone rich in citrus), the flavonoid baicalein, the carotenoid lycopene and CUR have produced positive outcomes in in vitro and in vivo studies. Indeed, it has been demonstrated that these compounds can act on mitochondrial integrity, ATP production, mitochondrial membrane potentials, and GSH levels, halting increased ROS production and apoptosis and mitigating altered mitochondrial mechanisms [85,86,87,88,89,90,91].

In our study, after 21 days of treatment, DHZ-DIM proved to be more effective than monomer in reducing mitochondrial damage in LRRK brain flies, suggesting that DHZ-DIM can suppress mitochondrial dysfunction, as already demonstrated for the above antioxidants.

Although numerous studies in cell and animal models support the potential of antioxidants in treating PD, many of these results cannot be reproduced in humans.

As a matter of fact, clinical trials have not demonstrated any efficacy of creatine or coenzyme Q10 in patients with PD [92,93]. A possible explanation for these negative clinical results is that oxidative stress could be a downstream effect of mitochondrial dysfunction rather than a direct cause of PD neurodegeneration. Otherwise, of novel drug-delivery approaches may be required.

Finally, we noticed a recovery in the loss of T-bars in the mutant flies after the chronic treatment with DHZ, which was more significatively pronounced with DHZ-DIM. T-bars are the presynaptic active zones involved in neurotransmitter release in Drosophila [94]. LRRK2 binds synaptic vesicles through specific protein–protein interactions in the WD40 domain [95]. Remarkably, synaptic proteomic analysis showed that the G2385R variant impairs LRRK2 binding to key synaptic proteins, including synapsin, which may explain the loss of T-bars in the LRRK2 WD40 Drosophila model [49]. Moreover, evidence supports the role of mitochondria in synaptic plasticity by maintaining cytosolic calcium within physiological ranges [96]. Therefore, a reduced mitochondrial antioxidant function could also be linked to synaptic loss. The neuroprotective effect of DHZ-DIM could be related to its capacity to prevent mitochondrial damage and synaptic loss by its antioxidant activity.

## 5. Conclusions

In summary, the overall findings from this study indicated that DHZ-DIM, more than its monomer, possessed a strong neuroprotective effect through its ability to ameliorate the PD-like phenotypes in our Drosophila PD model. If validated in mammalian models of PD, it could be considered a promising compound for the design and development of novel nutraceutical agents with neuroprotective properties to be evaluated in humans. However, it should be also considered both that the additional demands to counteract pathological conditions and the practical inability to provide dietary intake with the necessary “overdose” of nutraceuticals present only in traces in available aliments is possibly decisive for a real impact against neurodegenerative diseases.

Although there is strong evidence that suggests to what extent dietary intake of natural compounds with antioxidant and anti-inflammatory properties may inhibit neurodegeneration in PD, more detailed studies are needed exploiting alternative neurodegenerative disease models in both mammals and Drosophila in combination with clinical trials. These further experiments will contribute to providing hope for future effective therapies against PD with natural compounds.

## Data Availability

The data presented in this study are available on request from the corresponding author.

## Supplementary Materials

The following supporting information can be downloaded at https://www.mdpi.com/article/10.3390/biom14030273/s1. Figure S1: Arrows indicate (a) red abdomen (DHZ) and (b) blue abdomen (DHZ-DIM) of LRRK Drosophila reared on medium food dye compared with an LRRK on standard food (c).
