# Supplementary material for: Neuroprotective Effects of the Nutraceutical Dehydrozingerone and Its C2-Symmetric Dimer in a Drosophila Model of Parkinson’s Disease
Source: Biomolecules. 2024 Feb 24;14(3):273. doi: 10.3390/biom14030273 (PMC10968126; doi:10.3390/biom14030273)
Supplement: Supplementary file 1 [file biomolecules-14-00273-s001.zip › biomolecules-2811733-supplementary.pdf]

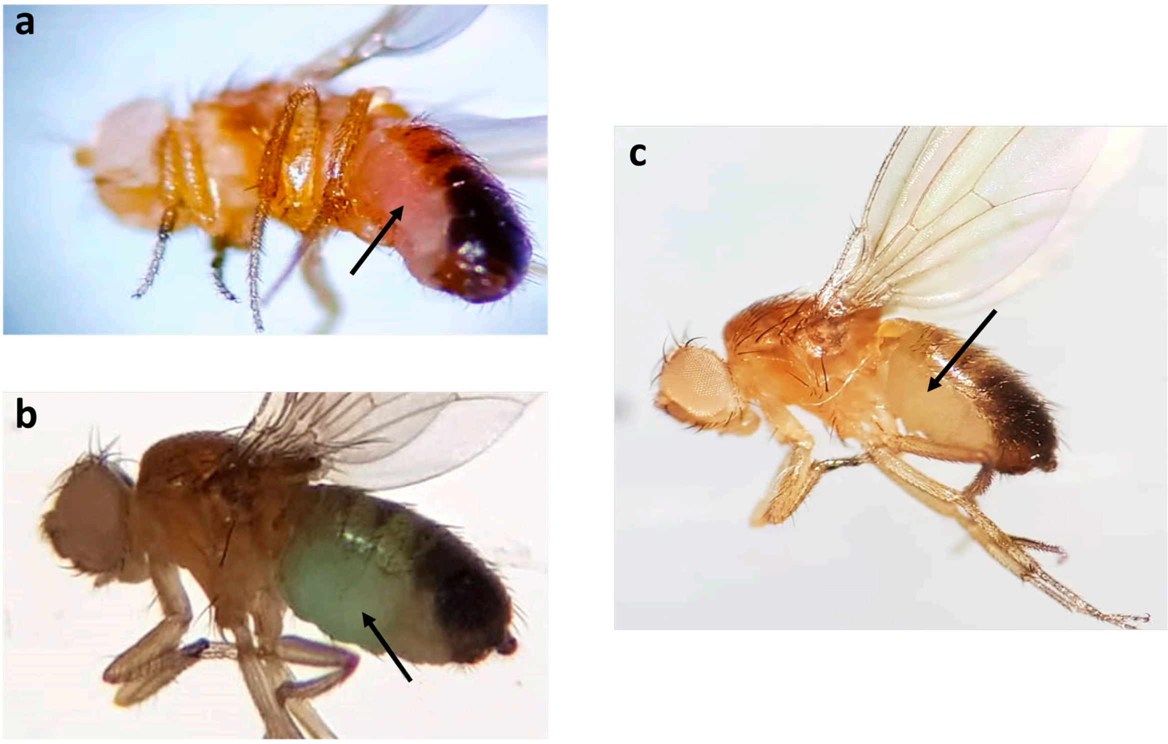

Figure S1. Arrows indicate a) red abdomen (DHZ) and b) blue abdomen (DHZ-DIM) of LRRK Drosophila reared on medium food dye compared with a LRRK on standard food (c).
